# Analysis of Characteristics and Quality of Life of Elderly Women with Mild to Moderate Urinary Incontinence in Community Dwellings

**DOI:** 10.3390/ijerph19095609

**Published:** 2022-05-05

**Authors:** Di Zhang, Shiyan Wang, Lei Gao, Yuanyuan Jia, Haibo Wang, Xiuli Sun, Jianliu Wang

**Affiliations:** 1 Department of Obstetrics and Gynecology, Peking University People’s Hospital, No. 11, Xi-Zhi-Men South Street, Xi Cheng District, Beijing 100044, China; 1911110388@bjmu.edu.cn (D.Z.); 0062043910@bjmu.edu.cn (S.W.); 1911110389@bjmu.edu.cn (L.G.); jiayuan@bjmu.edu.cn (Y.J.); wangjianliu@pkuph.edu.cn (J.W.); 2 The Key Laboratory of Female Pelvic Floor Disorders, Beijing 100044, China; 3 Research Center of Female Pelvic Floor Disorders, Peking University, Beijing 100871, China; 4 Clinical Research Institute, Peking University, Beijing 100871, China; wanghb_pucri@bjmu.edu.cn

**Keywords:** urinary incontinence, mild and moderate, quality of life, sex life

## Abstract

Introduction: The incidence of urinary incontinence (UI) increases with age. Mild and moderate UI have little impact on women and are easily ignored. This study reports the characteristics of non-severe UI and quality of life (QOL) in elderly women using data from a Development and Evaluation of a Senile UI Alert System study. Methods: 926 women aged ≥60 were enrolled from six subcenters across China, among whom 717 SUI patients and 209 UUI/MUI patients were grouped into Group A and Group B, respectively, according to leakage symptoms. Demographic and clinical data, pelvic organ prolapse quantification and pelvic floor muscle strength measurement (PFMS) were collected from participants, followed by evaluation of QOL and sexual life. Result: The major type of UI in community women was SUI (77.4%); MUI and UUI accounted for 20.63% and 1.94%, respectively. Weakened PFMS was detected in 78.2% of the participants. Group B was significantly higher in terms of median age, weight, BMI, waist circumference and menopausal years, and had the greater UI severity and impact on QOL, as well as less active sex live than group A (*p* < 0.05). Conclusion: SUI distributes dominantly in elderly community women, but UUI/MUI has greater impact on QOL and is related to a less active sex life, which requires more attention from medical staff.

## 1. Introduction

UI is a common health problem and defined by the International Continence Society (ICS) as a lower urinary tract symptom associated to the complaint of involuntary leakage of urine from the urethral opening [1]. UI occurs in all ages, especially in middle-aged and elderly women; 14.1% to 68.8% of adult women suffer from UI [2] and the incidence of UI is estimated to be 38% in women over 60 years old [3,4], which increases with age. UI is categorized into three types based on etiology. Stress urinary incontinence (SUI) is the most common type of UI accounting for 50% of all [5], followed by mixed urinary incontinence (MUI) with symptoms of urgent leakage of urine under stress, and urgency urinary incontinence (UUI) [6]. The prevalence reported by studies varies from 1% to 7% for UUI and from 7.5% to 25% for MUI [5].

UI can be mild to severe according to the symptoms in reference to the Ingelman–Sundberg classification [7,8] and the International Continence Society standards [9]. Many elderly women have a UI. Since mild and moderate UI are symptomatically unobvious, they are usually not detected at a preventable stage due to a lack of recognition of UI, misconception of UI and embarrassment to search for medical care at the patient end [10], as well as insufficient attention at the provider end to elderly women with mild and moderate UI. Once UI progresses to moderate or severe, it will heavily affect a patient’s life in social, psychological and physical aspects in several ways [11,12,13]. Although not a life-threatening disease, UI, especially severe UI, can cause prolonged disruption in women’s lives. Some clinical studies have indicated that symptomatic severity of UI has an obvious effect on quality of life (QOL); women with severe UI often have poorer QOL than those with mild UI or without UI [14,15].

The classic definition of QOL was described by Felce et al. in 1995. They believed that QOL is a wide-ranging concept and defined it as the overall general wellbeing that comprises the objective evaluation of physical and material health, social relationships and emotional wellbeing together with the extent of an individual’s development, group activities and subjective descriptions of personal satisfaction with the above domains of life [16]. As a common genitourinary tract disease, UI affects women’s QOL a lot, especially their sexual life. Many studies have shown a decrease in sexual wellbeing of women with UI [17,18]. Its great impact on sexual life negatively impacts the women’s sexual relationships, the most important social relationship as a determinant of QOL, and the objective description of the emotion, another determinant of QOL.

To prevent UI from progressing to be severe and bringing poor QOL, it is essential for clinicians to provide interventions for women’s QOL as early as possible. Preventive interventions are based on identification of the affecting risk factors and comprehension of the impact of mild to moderate UI on women.

Against this background, we designed a prospective multicenter cohort study using Development and Evaluation of a Senile UI Alert System (DESUIAS), which was previously titled Construction of Progress Prediction Model of Urinary Incontinence in Elderly Women when its protocol was published before it was amended to the current name. Being implemented in China, DESUIAS was projected to develop an effective UI progress prediction model based on the risk factors related to UI progress to be identified via analysis of the participants’ data collected in the baseline survey and follow-ups. The objective of this manuscript is to report the characteristics of the participants and the impacts of UI on their QOL based on the analysis of the baseline data collected from the participants that have been enrolled in DESUIAS up to now.

DESUIAS was reviewed and approved by the Ethics Review Board of Peking University People’s Hospital (2020PHB054-01) and the protocol was published in the International Journal of Environmental Research and Public Health [19].

## 2. Materials and Methods

DESUIAS was launched in May 2020 and is being continued to December 2022. Study centers were set up in Peking University People’s Hospital in Beijing and Wuhan University People’s Hospital in Wuhan, China, and an additional six cooperative hospitals from Beijing and Wuhan were involved in the study as the subcenters of the two study centers. Women ≥60 years of age were approached by investigators of the subcenters from communities near the subcenters via phone calls and they were primarily screened by inquiring whether they were suffering any urgent leakage or abdominal pressure leakage, how frequent the leakage was and if any symptoms were accompanying the leakage. Based on the women’s responses to the phone call inquiries, women who were suspected of having potential mild and moderate UI from the primary screening or those previously diagnosed with UI, no life-threatening diseases such as severe cardiovascular and cerebrovascular diseases and malignant tumors, no history of anti-UI surgery and medication for UI, and who were educated and mobilizable enough for completing the questionnaires and follow-up visits were eligible for participation and were invited to visit the nearby subcenter for questionnaire interview and confirmative pelvic floor examination as the secondary screening.

Participant recruitment was conducted in gynecological clinics of all the subcenters. Eligible women who visited the subcenters were enrolled (the participants) if they agreed to participation by providing a signed consent form. Participants were excluded if they were clinically confirmed to have severe pelvic organ prolapse (POP), urogenital fistula, severe UI, chronic urinary retention or severe cardiovascular and cerebrovascular diseases and malignant tumors in the questionnaires, interviewing and pelvic examinations. In order to ensure the quality of the study implementation, including participant interview and pelvic examination, all the subcenter investigators were trained prior to the launch of the study and procedures were regularly inspected by the principal investigator from the Peking University People’s Hospital.

After registration, each participant was given a study ID and then led to a private room for a baseline data inquiry about their demographic information, their obstetric, menopausal, chronic disease and UI history, the impact of UI on quality of life (QOL) and their sexual activities, followed by interviewing to complete the Overactive Bladder Symptom Score (OABSS) questionnaire and the International Consultation on Incontinence Questionnaire-Short Form (ICIQ-SF). The interviews were conducted in a face-to-face manner between just the interviewer and the interviewee. Pelvic examinations were then performed, in which pelvic floor muscle strength (PFMS) was measured and pelvic organ prolapse quantification (POP-Q) was detected. The type and severity of the participants’ UI were confirmed by the investigators in reference to the urinary leakage symptoms, ICIQ-SF and OABSS outcomes. Participants were informed about their clinical diagnosis for UI as well as the UI type and severity and the scheduled follow-up visits, which were conducted every 24 weeks three times. As we have not analyzed the data from the follow-ups in this manuscript, details of these follow-ups will be introduced in a deferent manuscript.

**Figure 1:**
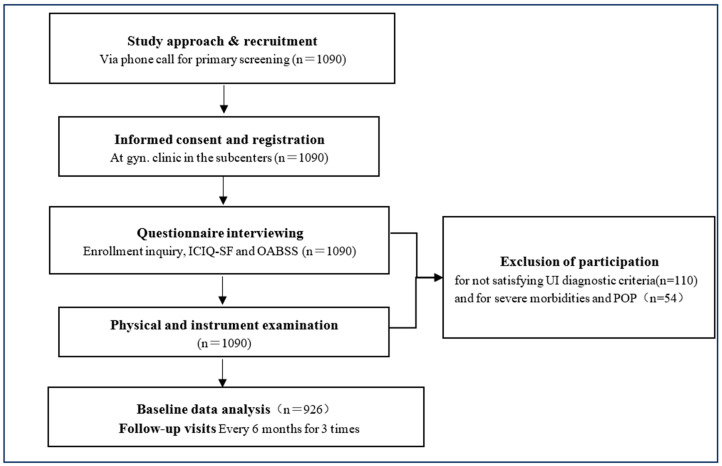
Flow chart for participation.

The flow chart of DESUIAS related to this analysis is as follows (Figure 1):

### 2.1. Measurement

The questionnaire for enrollment covers demographic information including the woman’s age, educational level and lifetime dominant occupation; obstetric history in terms of delivery methods (vaginal delivery, cesarean section or both, and delivery with episiotomy) and maximum fetal weight; the occurrence of symptomatic urine leakage during pregnancy and postpartum; the family history of UI; drinking habits referring to beverage preference (tea/water, commercial beverages or other diuretic drinks) and usual volume of liquid intake every 24 h; menopausal information on initial age, mode (spontaneous or surgical) and postmenopausal years; and chronic disease status in terms of chronic cough, asthma, diabetes mellitus, constipation and depression.

Data regarding to the sex life of the participants were also collected with two simple and comprehensible questions: (1) whether they had had sex with their partner(s) in the previous 12 months; and (2) what was the best description of their evaluation of their sexual life followed by several choices such as “very satisfied”, “satisfied” and “unsatisfied”; a participant was identified as “sexually active” if she “had sex with at least one partner in the previous 12 months” [20]. Sexual satisfaction for a specific participant was attributed by the investigators based on the overall assessment of the patient’s subjective feelings to the positive and negative aspects of their sexual relationships [21].

ICIQ-SF is a brief and simple questionnaire that can be self-administered. It is highly recommended as the questionnaire for investigation of the presence of symptomatic UI and their impact on QOL, and is widely used in research for its high reliability. Several high-quality randomized clinical trial (RCT) studies applied this questionnaire to assess the severity of UI and QOL in women [22,23,24]. The Chinese version of ICIQ-SF is consistent with the English version and has been validated by Liang Huang et al. [25]. It is composed of four items to evaluate the frequency and volume of urine leakage and offers a quick evaluation of the impact of UI on QOL via scoring from 0 to 10 to represent qualitative degrees of the impact: 1 for “no impact” and 1 to 3, 4 to 6, 7 to 9 and 10 for mild, moderate, severe and extensive, respectively [26]. The score summarizing the first three items gives the score for UI severity, which ranges from 0 to 21 [24,27]; the higher the score, the higher the severity [27]. Also included on ICIQ-SF are eight self-diagnosis items to investigate the UI situation experienced by women: (1) never leaking urine; (2) leaking urine before reaching the toilet; (3) leaking urine while sneezing or coughing; (4) leaking urine while sleeping; (5) leaking urine during activity or exercises; (6) leaking urine after urinating or getting dressed; (7) leaking urine with unapparent reason; (8) leaking urine at all times. Women were primarily diagnosed as no-UI if item (1) was chosen, SUI if (3) and/or (5) were chosen, UUI if (2), (4) and/or (6) were chosen, and MUI if (7) and/or (8) were chosen [27]. This result will be referred to for participant grouping in the data analysis.

The OABSS questionnaire is intended to assess the symptoms of an overactive bladder and the severity of UUI. It evaluates daily voiding frequency, nocturia, frequency of urgent urination and urge incontinence by scoring 0–15 for each of the four items, with <2 for unurgent UI, 3–5 for mild, 6–11 for moderate and 12–15 for severe UUI [28].

Following the questionnaires is the pelvic examination, in which measurements of women’s heights, weights, body mass indexes (BMI), waist and hip circumferences and waist-to-hip ratio (WHR) were conducted by the gynecologists. POP-Q system was performed [29] and PFMS was assessed by digital palpation and scored according to the Modified Oxford Scale (MOS) from 0 to 5 (0 = no muscle contraction detected; 1 = slight muscle contraction; 2 = weak muscle contraction; 3 = moderate muscle contraction; 4 = good muscle contraction with lift; 5 = strong muscle contraction with good lifting effect) [30]. PFMS was defined as normal if MOS equaled 4–5 or weak if MOS equaled 0–3 [31,32].

SUI was scaled based on the symptoms confirmed using the Ingelman–Sundberg classification [7,8], according to which, mild refers to UI that occurs during coughing or sneezing without the need for urinal pads; moderate refers to UI that occurs when running, jumping or fast walking, with urinal pads needed; and severe refers to UI that occurs with body-position changing.

MUI was scaled to match the higher symptomatic scales for SUI or UUI because patients with MUI have both SUI and UUI symptoms. Accordingly, the scale of MUI is the higher symptomatic scale between that of SUI and UUI on the same woman.

We divided UI patients into two groups: Group A for SUI (urine leakage under abdominal pressure) and Group B for UUI/MUI (urgency leakage). We grouped UUI and MUI together because our MUI patients, despite having both SUI and UUI symptoms, were mostly urgency leakage dominant.

### 2.2. Statistical Methods

Using descriptive analysis, this manuscript summarized the demographic and clinical data of participants. Data analysis was performed using version 23.0 of IBM SPSS. Continuous variables were analyzed with the Shapiro–Wilk test. Variables were analyzed with independent sample t-tests and presented as the mean and standard deviation (SD) if they were normally distributed. Variables were analyzed with Mann–Whitney U tests and presented as the median and interquartile range (25th quartile and 75th quartile) if they were not normally distributed. Pearson’s chi-squared test was used to analyze the difference in the categorical variables between Group A and Group B, and the grading data were analyzed using the Mann–Whitney U test. Categorical and grade variables were described using absolute and relative frequencies. Variables between the two groups were considered statistically significant if *p* < 0.05.

## 3. Results

To date, 24 communities have been approached about the project and 1090 women who were identified eligible for participation visited the subcenter and signed informed consent forms. After 54 women were excluded due to clinically confirmed contraindications and 110 women were excluded for not satisfying UI diagnostic criteria through the ICIQ-SF and OABSS questionnaires, 926 women were enrolled in the study as the participants, with a mean age of 65.6 (60–89) years. Via questionnaires and pelvic examinations, 717 (77.43%) participants were confirmed SUI, 18 (1.94%) were UUI and 191 (20.63%) were MUI. A predominant part of SUI cases (90.9%, 652/717) were symptomatically graded mild; and 83.3% (15/18) of UUI cases and 89% (170/191) of MUI cases were symptomatically moderate. (Table 1).

As shown in Table 2, Group B significantly exceeded Group A in median age, weight, BMI, waist circumference and menopausal years (*p* < 0.05). Group A showed a higher proportion of cases with ≥3 parity than Group B (*p* < 0.05). Constipation, diabetes mellitus, asthma and depression were identified in both groups with rates of 28.9%, 13%, 4.2% and 1.7% in Group A and 33%, 12.9%, 7.2% and 1.9% in Group B, respectively, but with no significant difference. A statistically significant difference between the two groups was found for chronic cough, with a prevalence of 13.2% for group A and 25.4% for Group B (*p* < 0.05). There were no significant differences in other variables; however, Group A showed a slightly higher rate of vaginal delivery, episiotomy and natural menopause than Group B. Regarding drinking habits, the two groups were not statistically different from each other in beverage preference and volume of liquid intake in 24 h (*p* > 0.05).

Weakened PFMS were found in 78.2% of the participants (all with UI) and there was no statistically significant difference between the two groups regarding POP measurement and PFMS assessment, indicating that muscle strength commonly decreased in elderly women (Table 3).

**Figure 2:**
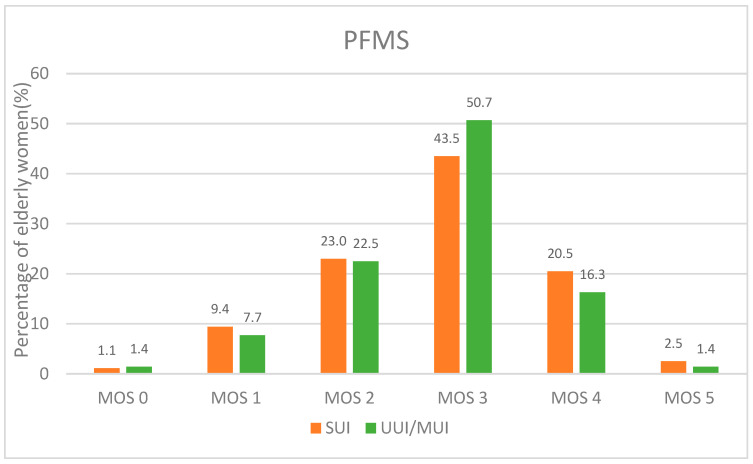
PFMS scores by MOS for the participants.

Figure 2 shows the distribution of MOS scores for PFMS in the two groups. It is obvious that the majority of participants in both groups were scored MOS = 3, followed in up-down ranking by MOS = 2 and MOS = 4, with no significant difference in all the MOS scores. Again, the fact that only a limited number of UI patients were scored MOS = 0 and 5 indicates commonly weakened PFMS among elderly women (Figure 2).

The overall ICIQ-SF median score of Group B was 7 (5–10), significantly higher than that of Group A, which was 5 (4–6) (*p* < 0.001). Analysis of UI impact on participants’ quality of life (QOL) via ICIQ-SF questionnaires showed that 14% of elderly women with non-severe UI did not think UI had an impact on QOL by choosing “0, not at all”, while 86% of women felt that UI had an impact on QOL to some extent. The mean score of Group B was 3 (1–5), significantly higher than the same score of Group A, which was 2 (1–3) (*p* < 0.001); 112 of the 717 (15.6%) participants in Group A and 17 of the 209 (8.1%) in Group B believed that that UI had no negative impact on their QOL; 65.6% (470/717) and 53.6% (112/209) of those in Group A and B, respectively, attributed a “mild” level to the UI impact on their QOL, but “moderate” and greater levels of impact on QOL were marked by 38.3% (80/209) of the participants in Group B and 18.8% (135/717) of those in Group A. In addition, Group B was higher than Group A in the ratio of women choosing all levels of impact from moderate to extensive. Those results indicate that UI impact on elderly women with UUI/MUI is generally greater than that on the same-aged women with SUI (Table 4).

Being sexually active was attributed to a woman if she responded to the inquiry with “having sex with at least one partner in the past 12 months”. Among all the participants, 30.6% (283/926) were reported sexually active, while 69.4% (643/926) chose “sexually inactive”. Grouping analysis showed that the two groups were significantly different in the proportions of women who were sexually active or inactive (*p* < 0.05). Table 5 shows that Group A was higher than Group B in the proportion of sexually active women (32.2% (231/717) vs. 24.9% (52/209), and 67.8% (486/717) and 75.1% (157/209) of the participants in Group A and Group B, respectively, were sexually inactive in the past 12 months. Due to only 1/3 of the participants being sexually active, we failed to observe a significant difference between the two groups in the qualitative choices for “very satisfied”, “satisfied” or “unsatisfied” (*p* = 0.050). However, data on the feelings of the participants on their sexual lives still showed that 91.9% (260/283) of all the women who reported being sexually active felt satisfied or above with their sexual life (Table 5). Those results suggest that, regarding less statistical difference, UUI/MUI patients had a lower rate of active sex lives than SUI patients, but that there were no statistical differences in the satisfaction of elderly women who are sexually active.

## 4. Discussion

Based on the analysis of the data of the participants from DESUIAS, we concluded that a big portion of elderly community women who were suffering with UI were symptomatic SUIs. Symptoms of UUI/MUI have a greater impact than those of SUI on the QOL and sexual life of elderly women.

Since this manuscript is focused on analyzing the characteristics of UI and the impact on the QOL of elderly women, we will analyze the risk factors for UI progression and establish a DESUIAS in an individual publication in the future.

Our finding that the prevalent ranking of UI among elderly community women with UI are SUI (77.43%), MUI (20.63%) and UUI (1.94%) is similar to the reported findings by Xu in China, which is targeted at Chinese women aged 50–70 years [27]. However, the reported prevalent rank of the three UI subtypes differs among several studies on different ages of women. Those results suggest that UI types may be likely transitable from one to another by remission or progression along with ageing, which should be evidenced by years of follow-up [33].

Many factors have been reported to be potentially associated with UI to date, including age, BMI, genetic factors, constipation, pregnancy and multiple childbirths, pelvic surgery and menopause [34]. Our study compared mild and moderate SUI and UUI/MUI in elderly women in groups, and found that the UUI/MUI (Group B) patients had higher mean age, more years of postmenopause and greater values on obesity-related indicators including weight, BMI and waist circumference than SUI patients. A white cohort study [35] on older women aged 54 to 79 with UI from the Nurses’ Health Study found that BMI was related to UUI and MUI; however, waist circumference was a predictor for SUI. Our study demonstrated a difference to the conclusion from that study, which may be associated with the different ethnicity of the participants. We also found that chronic cough in elderly women with UI was a statistically significant indicator of SUI and UUI/MUI. A review by Xue [34] found that several chronic diseases were associated factors of UI. However, our study represented a different prevalence of chronic disease in subtypes of UI, which could be of great significance to mitigate the different subtypes of UI incidence by treating chronic diseases.

In this study, we found that the PFMS of elderly women with UI was generally weakened, with supportive facts that 78.2% of patients had weakened PFMS and the majority of muscle strength grades were grade III and grade II. As reported, the pelvic floor muscles may become weakened by aging, delivery or surgery. Elderly women may easily suffer from UI and pelvic organ prolapses due to the weakening of the pelvic floor supportive system, and a decrease in QOL is also confirmed [36,37]. To our knowledge, there are few studies comparing PFMS and POP with subtypes of UI. In this study, we discovered that there was an insufficiency of statistically significant differences for PFMS and POP among SUI and UUI/MUI, which may be related to all the participants with non-severe UI.

The relationship between UI and QOL has been scientifically and clinically confirmed over the years. UI decreased quality of life in older women, and increased fractures, falls and nursing home admission [38]. Therefore, in addition to the comparison and description of the disease characteristics of these two groups above, our study mainly evaluated the overall QOL of women with non-severe UI.

The ICIQ-SF questionnaire was used to investigate the disease’s severity and general impact on QOL. In our study, 86% of elderly women confirmed UI’s negative impact on their lives. Comparison of the ICIQ-SF outcomes of SUI and UUI/MUI patients shows that UUI/MUI patients suffered from a greater severity of UI and worse impact on their life. Several studies on this topic have demonstrated that UI has certain impacts on women’s life; however, debate exists on which type of UI has the greatest impact on QOL of UI patients. Our study found that urgent urine leakage of UUI and MUI had worse impact on QOL than SUI, which is similar to Åström et al. [39] who mentioned that the QOL of women with urgency and mixed UI was affected more than that of women with stress UI by ICIQ-LUTSqol score assessment. Bunyavejchevin et al. [40] and Coyne et al. [41] also demonstrated results by different QOL questionnaires that are consistent with our study. Furthermore, Bunyavejchevin reported that women with MUI had lower health than OAB in all domains by SF-36 scores, but Coyne failed to find any differences in the impact of MUI or UUI on QOL by HRQL scale. A review conducted by Basak et al. [42] from Turkey summarized 20 studies related to the QOL of UI women and indicated that MUI and SUI were reported in four and two studies, respectively, to have more effects on the QOL than the other subtype of UI. This result resembles studies in other countries. From the above studies, urgency incontinence (MUI or UUI) may be closely associated with a higher grade of impact on women’s QOL; those women are more likely to experience functional limitations and declining wellbeing from UI.

UI, as one of the diseases of the genitourinary system, also has a negative effect on women’s sexual experiences. With incontinence or without incontinence during sexual activity, UI often decreases the sexual desires and wellbeing of the women [43]. Evaluation of sexual life should be a vital component of QOL since sexual dysfunction is associated with QOL and sexual situation is closely tied to women’s self-worth, depression, loneliness and cognitive function [44].

Our QOL evaluation of UI patients was mainly focused on the overall sexual life situation. From analysis of this sex domain, we found that only 30.6% of elderly women with UI were sexually active. A survey in the United States reported that the prevalence of sexual activity among 1550 women and 1455 men aged 57–64, 65–74 and 75–85 were 73%, 53%, and 26%, respectively, declining as age increased [20]. Stadnicka [45] showed that, in a study covering 275 SUI women, 68.7% engaged in sexual activities, but the age span was 30–65 years. Compared with the above studies, UI patients were generally less sexually active in our study than in women with or without SUI in other reported studies. The difference may lie in the fact that the participants included in our study were all 60 to 89 years of age with a comparatively lower activity of sex life. Nonetheless, it is reasonable to believe that UI, as the result of PFM weakness, negatively impacts elderly women’s sexuality.

Most studies found that certain subtypes of UI were correlated with sexual dysfunction profiles [38]. In our study, UUI/MUI patients had poorer QOL, a lower rate of active sex life and a higher rate of dissatisfaction with sex than SUI patients, although no significance in satisfaction. The MUI patients we enrolled were urgency leakage dominant, with slight SUI symptoms. We concluded that the sex life of the patients with UUI/MUI were negatively impacted much more than that of patients with simple SUI. Multiple studies have found consistent results with us; that is, UUI or MUI affected sexual function more than SUI in many domains of sex activity. Karbage et al. [46] reported that MUI affected sexual function more than SUI, which is in line with the results reviewed by Duralde [38] who summarized that MUI was associated with the greatest sexual dysfunction including less sexual activity and decreased satisfaction and desire, followed by UUI, and finally SUI.

The reason why UUI and MUI have a more serious impact on sex than SUI lies in the etiology and pathophysiology. Compared with SUI women, UUI women more likely need to urinate before and after sexual activity due to the unpredictable and unavoidable urgency caused by an overactive bladder [47]. MUI refers to a combination of UUI and SUI symptoms. It is not difficult to reach this conclusion that MUI has a greater impact on sexual life than simple SUI [38].

Our data could not confirm whether satisfaction with sex life was associated with the type of UI in elderly women; this result was unexpected. The association between sexual activity and sexual satisfaction is complex, which may be directly related to women’s sexual satisfaction in younger ages when they had no UI [44] or their different perceptions and expressions of satisfaction with sex at older ages.

Evaluation of sexuality as an important part of women’s quality of life shows that a lower rate of sexual life was associated with a lower quality of life. It is posited that reducing the symptoms of urinary leakage may help UI women improve sexual situations and achieve a better quality of life. Some studies have addressed the importance of treating UI and changes in sexual function, including improvement of sexual desire, arousal and orgasm [48,49].

Elderly women who have a non-severe UI, especially a minority of women with UUI and MUI, who are suffering from a worse QOL, need more attention and recommendations from medical staff before progressing to severe UI. Recommendations should include losing weight, treatment of chronic diseases, regularly pelvic floor muscle exercise to enhance muscle strength [37], recognition and amelioration of potential barriers to healthy sexual expression and any others that can improve QOL and sex life for older women and their loved ones.

Furthermore, elderly women usually do not have sufficient knowledge about UI. Thus, it is essential to give them adequate information and instruction. The community health center and district-level care hospital should use plain language to avoid medical terms to educate elderly women with mild or moderate UI, particularly with UUI and MUI, to comprehend the harm of UI on their health and life and encourage them to see a doctor early. In the future, UI progress prediction models will be further constructed to look for risk factors for progress, and some intervention measures will be taken to target these risk factors and better improve their QOL.

As our analysis on the UI impacts on QOL is based on the enrollment data of DESUIAS, the analysis has certain limitations. First, considering elderly women’s embarrassment, shame and misunderstanding of professional issues on sex, we preliminarily evaluated their sex life by a two-point question regarding their sexuality and sex-related subjective feelings of satisfaction. The pilot investigation before our study showed as well its understandability to elderly women. We found UI patients had a poor sexual situation. However, to achieve better outcomes, elderly women are recommended to answer more questions on sexual dysfunction, including sexual desire, arousal, lubrication and orgasm by professional sexual dysfunction questionnaires after acquiring explanation from medical staff in the future. Second, our results showed significant differences between the SUI and UUI/MUI groups; participants from the community in this study were recruited by the convenience sampling method. Due to the different prevalence of different types of UI, there were fewer cases of MUI and UUI than SUI. Future studies are needed with more cases of subtypes of UI to confirm the differences between QOL and urgency leakage and stress leakage of urine.

## 5. Conclusions

In conclusion, SUI is the most common type of UI among elderly women in the community and UUI/MUI exerts more adverse effects on women’s quality of life with a less active sex life, thus suggesting that clinicians should pay more attention to the mild and moderate UI, especially UUI/MUI regardless of the comparatively lower incidence, to avoid UI progression.

## Figures and Tables

**Table 1:** UI type distribution.

	Group A		Group B			
Total (n)	SUI (n = 717/77.43%)		UUI (n = 18/1.94%)		MUI (n = 191/20.63%)	
	Mild	Moderate	Mild	Moderate	Mild	Moderate
926	652 (90.9%)	65 (9.1%)	3 (16.7%)	15 (83.3%)	21 (11.0%)	170 (89%)

SUI: stress urinary incontinence; UUI: urgency urinary incontinence; MUI: mixed urinary incontinence.

**Table 2:** Demographic and clinical data of the participants in the two groups.

	Group A (SUI) (n = 717)	Group B (UUI/MUI) (n = 209)	Z/χ^2^	*p*
Age (years)	64 (62–68)	66 (62–69)	−2.686	0.007 * ^a^
Educational level			−0.198	0.843 ^a^
Primary education or less	224 (31.3%)	68 (32.6%)		
Secondary education	424 (59.1%)	120 (57.4%)		
Tertiary education or above	69 (9.6%)	21 (10%)		
Lifetime dominant occupation			−0.545	0.586 ^a^
Mental activity	115 (16%)	37 (17.7%)		
Grade I mild ^1^	182 (25.4%)	46 (22%)		
Grade II moderate ^2^	362 (50.5%)	103 (49.3%)		
Grade III heavy ^3^	56 (7.8%)	23 (11%)		
Grade IV extreme heavy ^4^	3 (0.3)	0 (0%)		
Height (cm)	158 (155–161)	158 (155–160)	−0.527	0.598 ^a^
Weight (kg)	62 (56–68))	63 (58.25–70.0)	−1.984	0.047 * ^a^
BMI (kg/m^2^)	24.7 (22.6–26.7)	25.4 (23.00–27.5)	−2.397	0.017 * ^a^
Waist circumference (cm)	90 (85–97)	93 (86–100)	−2.087	0.037 * ^a^
Hip circumference (cm)	100 (95–105)	101 (97.0–106.5)	−1.590	0.112 ^a^
Waist-to-hip ratio (WHR)	0.9 (0.87–0.95)	0.91 (0.88–0.95)	−1.077	0.281 ^a^
Delivery mode			2.451	0.294 ^b^
VD	655 (91.4%)	186 (89%)		
CS	57 (7.9%)	19 (9.1%)		
Both	5 (0.7%)	4 (1.9%)		
Delivery with episiotomy	227 (31.7%)	58 (27.8%)	1.264	0.531 ^b^
Parity			5.379	0.020 * ^b^
0–2	550 (76.7%)	176 (84.2%)		
≥3	167 (23.3%)	33 (15.8%)		
Maximum fetal weight (kg)	3.20 (3.00–3.50)	3.25 (3.00–3.60)	−1.462	0.144 ^a^
Leakage of urine during pregnancy	42 (5.9%)	12 (5.7%)	0.309	0.857 ^b^
Leakage of urine postpartum	61 (8.5%)	18 (8.6%)	0.013	0.993 ^b^
Family history of UI	66 (9.2%)	29 (13.9%)	3.835	0.147 ^b^
Beverage preference	142 (19.8%)	33 (15.8%)	1.702	0.192 ^b^
Volume of liquid intake (24 h, mL)			1.828	0.401 ^b^
<1000	216 (30.1%)	57 (27.3%)		
1000–2000	374 (52.2%)	120 (57.4%)		
>2000	127 (17.7%)	32 (15.3%)		
Menopausal age	50 (48–53)	50 (48–52)	−1.100	0.271 ^a^
Menopause mode			1.867	0.172 ^b^
Nature	680 (94.8%)	193 (92.3%)		
Surgery	37 (5.2%)	16 (7.7%)		
Postmenopausal years	14 (11–20)	16 (12–21)	−2.863	0.004 * ^a^
Comorbidities				
Chronic cough	95 (13.2%)	53 (25.4%)	17.671	<0.001 * ^b^
Asthma	30 (4.2%)	15 (7.2%)	3.135	0.077 ^b^
Diabetes mellitus	93 (13.0%)	27 (12.9%)	0.000	0.984 ^b^
Constipation	207 (28.9%)	69 (33.0%)	1.328	0.249 ^b^
Depression	12 (1.7%)	4 (1.9%)	0.000	1.000 ^b^

* *p* < 0.05; ^a^ Mann–Whitney U tests; ^b^ chi-squared tests. UI: urinary incontinence; BMI: body mass index; VD: vaginal delivery; CS: cesarean section; PFMS: pelvic floor muscle strength. ^1^ Manual work or light leg movement (e.g., typing, sewing, etc.); operation of the instrument, upper arm force-based assembly work. ^2^ Continuous movement of the hands and arms, e.g., sawing wood; arm and leg work (e.g., truck and other construction equipment transport operations); arm and trunk work (e.g., intermittent lifting of medium weights, weeding, fruit picking, etc.). ^3^ Work with arm and trunk loads (e.g., lifting heavy loads, mowing grass, digging, etc.). ^4^ Extremely strong physical activities such as digging and carrying with great intensity.

**Table 3:** PFMS and POP status in the two groups.

	Group A (SUI) (n = 717)	Group B (UUI/MUI) (n = 209)	Total	χ^2^	*p*
POP				0.123	0.726 ^b^
YES	464 (64.7%)	138 (66.0%)	602 (65.0%)		
NO	253 (35.3%)	71 (34.0%)	324 (35.0%)		
PFMS				2.675	0.102 ^b^
Normal	165 (23%)	37 (17.7%)	202 (21.8%)		
Weakened	552 (77%)	172 (82.3%)	724 (78.2%)		

^b^ Chi-squared tests; POP: pelvic organ prolapses.

**Table 4:** Severity and impact of UI on QOL in elderly women.

	Group A (SUI) (n = 717)	Group B (UUI/MUI) (n = 209)	Total (n%)	Z/χ^2^	*p*
ICIQ-SF total score (0 to 21)	5 (4–6)	7 (5–10)		−8.618	<0.001 * ^a^
Impact on QOL (0 to 10)	2 (1–3)	3 (1–5)		−5.425	<0.001 * ^a^
(0) not at all	112 (15.6%)	17 (8.1%)	129 (14.0%)		
(1–3) mild	470 (65.6%)	112 (53.6%)	582 (62.8%)		
(4–6) moderate	113 (15.8%)	68 (32.5%)	181 (19.5%)		
(7–9) severe	16 (2.2%)	10 (4.8%)	26 (2.8%)		
(10) extensive	6 (0.8%)	2 (1.0%)	8 (0.9%)		

* *p* < 0.05; ^a^ Mann–Whitney U tests; ICIQ-SF: International Consultation on Incontinence Questionnaire-Short Form; QOL: quality of life.

**Table 5:** Comparison of the general sex life of elderly women in Group A and Group B.

	Group A (SUI) (n = 717)	Group B (UUI/MUI) (n = 209)	Total (n%)	Z/χ^2^	*p*
Active sex life				4.105	0.043 * ^b^
Yes	231 (32.2%)	52 (24.9%)	283 (30.6%)		
No	486 (67.8%)	157 (75.1%)	643 (69.4%)		
Degree of satisfaction with active sex life				−1.958	0.050 ^a^
Very satisfied	69/231 (29.9%)	8/52 (15.4%)	77/283 (27.2%)		
Satisfied	144/231 (62.3%)	39/52 (75.0%)	183/283 (64.7%)		
Unsatisfied	18/231 (7.8%)	5/52 (9.6%)	23/283 (8.1%)		

* *p* < 0.05; ^a^ Mann–Whitney U tests; ^b^ chi-squared tests.

## Data Availability

Not applicable.

## Trial Registration

www.chictr.org.cn (Registration Number: ChiCTR2000031375) on 29 March 2020.
